# Podocalyxin Is a Marker of Poor Prognosis in Pancreatic Ductal Adenocarcinoma

**DOI:** 10.1371/journal.pone.0129012

**Published:** 2015-06-08

**Authors:** Kapo Saukkonen, Jaana Hagström, Harri Mustonen, Anne Juuti, Stig Nordling, Christian Fermér, Olle Nilsson, Hanna Seppänen, Caj Haglund

**Affiliations:** 1 Department of Surgery, University of Helsinki and Helsinki University Hospital, P.O. Box 440, FIN-00029 HUS, Helsinki, Finland; 2 Research Programs Unit, Translational Cancer Biology, University of Helsinki, P.O. Box 63, FIN-00014 University of Helsinki, Helsinki, Finland; 3 Department of Pathology, Haartman Institute and HUSLAB, University of Helsinki and Helsinki University Hospital, Helsinki, FIN-00014 University of Helsinki, Helsinki, Finland; 4 Fujirebio Diagnostics AB, Elof Lindälvs gata 13, SE-414 58 Gothenburg, Sweden; 5 Onson Consulting, Södra vägen 2, SE-412 54 Gothenburg, Sweden; Centro Nacional de Investigaciones Oncológicas (CNIO), SPAIN

## Abstract

**Aim of the Study:**

Podocalyxin-like 1 is a transmembrane glyco-protein whose overexpression associates in many cancers with poor prognosis and unfavorable clinicopathological characteristics. Until now, its prognostic value has never been studied in pancreatic ductal adenocarcinoma (PDAC). The aim of this study was to investigate podocalyxin expression in PDAC by a novel monoclonal antibody and a commercially available polyclonal antibody.

**Patients and Materials:**

With tissue microarrays and immuno-histochemistry, podocalyxin expression evaluation involved 168 PDAC patients. The associa-tions of the podocalyxin tumor expression with clinicopathological variables were explored by Fisher’s exact test and the linear-by-linear test. Survival analyses were by Kaplan-Meier anal-ysis and the Cox proportional hazard model.

**Results:**

The polyclonal antibody revealed membranous podocalyxin expression in 73 (44.0%) specimens and the monoclonal antibody was highly expressed in 36 (21.8%) cases. Membranous expression by the polyclonal antibody was associated with T classification (p=0.045) and perineural invasion (p=0.005), and high expression by the mono-clonal antibody with poor differentiation (p=0.033). High podocalyxin expression associated significantly with higher risk of death from PDAC by both the polyclonal antibody (hazard ratio (HR) = 1.62; 95% confidence interval (CI) 1.12-2.33; p=0.01) and the monoclonal antibody (HR = 2.10, 95% CI 1.38-3.20; p<0.001). The results remained significant in multivariate analysis, adjusted for age, gender, stage, lymph node ratio (≥/< 20%), and perivascular invasion (respectively as HR = 2.03; 95% CI 1.32-3.13, p=0.001; and as HR = 2.36; 95% CI 1.47-3.80, p<0.001).

**Conclusion:**

We found podocalyxin to be an independent factor for poor prognosis in PDAC. To our knowledge, this is the first such report of its prognostic value.

## Introduction

The prognosis of pancreatic ductal adenocarcinoma (PDAC) is extremely poor, with overall 5-year survival less than 5% [1,2]. Finland has about 1000 new cases per year, and PDAC is the third leading cause of cancer-related death [1]. In the United States, PDAC is the fourth leading cause of cancer-related mortality, causing annually about 40,000 deaths [2]. Despite improvements in management, the survival rate has not improved markedly over the last few decades [2]. At diagnosis, roughly 10% has localised disease and 25% regional disease [2]. Radical surgical resection is the only curative treatment, but disease is unresectable in about 80% of the cases [3]. Although overall survival is poor, there is a great variation between patients of the same stage, for whom new biomarkers might help in predicting prognosis. Research on potential biomarkers has been intense, but it still lacks good prognostic markers [4–6].

Podocalyxin-like 1 (PODXL), a transmembrane glycoprotein closely related to the hematopoietic stem cell marker CD34 and to endoglycan [7], regulates cell-to-cell adhesion through charge-repulsive effects but also contributes to cell morphology [7]. PODXL is normally expressed in hematopoietic progenitor cells [8], vascular endothelial cells [9], and renal podocytes [10]. First identified in the kidney, it helps there to maintain filtration pathways [11]. Loss of normal PODXL expression is associated with glomerulopathies—mainly with nephrotic syndrome [12].

PODXL overexpression has been described in several cancer types including leukemia and breast, colorectal, urothelial bladder, hepatocellular and prostate cancers [13–19]. In renal cell carcinoma, breast, and colorectal cancers, PODXL overexpression is an independent factor for poor outcome [14–16,20]. Whether PODXL is a marker of poor prognosis also in pancreatic cancer, remains unclear. It has been shown that PDAC cells are generally positive for PODXL, but other adenocarcinomas of the biliary and gastrointestinal tract are mainly negative [21]. Dallas et al. concluded that sialofucosylated PODXL is overexpressed by metastatic pancreatic cancer cells [22]. Furthermore, membranous PODXL expression has been suggested to correlate with poor prognosis in colorectal and urothelial bladder cancers [15–17]. Recently Kaprio et al. showed that high cytoplasmic expression of PODXL by a novel monoclonal antibody (mAb) is a marker of poor prognosis in colorectal cancer [23].

In this study we examined the prognostic value of PODXL expression in PDAC, comparing PODXL expression by our in-house HES9 mAb with a commercially available polyclonal antibody (pAb).

## Materials and Methods

### Patients

The study population comprised 189 consecutive PDAC patients surgically treated in 2000–2011 at the Department of Surgery, Helsinki University Hospital. Median age at operation was 64 (range 39–84) years. There were 21 patients, who had received neoadjuvant chemotherapy. Those were excluded from the study. Survival data and cause of death of the patients were obtained from patients’ records, Statistics Finland, and the Finnish Population Registry. This study complies with the declaration of Helsinki and was approved by the Surgical Ethics Committee of Helsinki University Central Hospital (Dnro HUS 226/E6/06, extension TMK02 §66 17.4.2013) and the National Supervisory Authority of Welfare and Health gave the permission to use tissue samples without individual informed consent in this retrospective study (Valvira Dnro 10041/06.01.03.01/2012).

### Preparation of tumor tissue microarrays

Formalin-fixed and paraffin-embedded surgical tissue samples were collected from the archives of the Department of Pathology, Helsinki University Hospital. Tissue specimens suitable for immunohistochemical evaluation were available from 168 patients. 15 patients were excluded due to inadequate or missing samples. All samples were re-evaluated by experienced pathologists (J.H. and S.N.) for confirmation of the histopathological diagnosis of PDAC. Most representative regions of tumor specimens were defined and tumor area was marked on hematoxylin- and eosin-stained tumor slides to prepare the TMA. Two 1.0-mm cores were taken from each tumor with a semiautomatic tissue microarrayer (Tissue Arrayer 1, Beecher Instruments Inc., Silver Spring, MD, USA).

### Antibodies

The novel monoclonal in-house antibody (HES9) used in this study recognizes amino acid residues 189–192 of PODXL. The polyclonal antibody (HPA 2110, Atlas Antibodies, Stockholm, Sweden) recognizes amino acid residues 278–415. The specificity of the polyclonal antibody has been validated by Western blotting and protein arrays, and PODXL protein expression mapped by immunohistochemistry in normal tissues and common cancers [24,25]. It has also served in other biomarker studies on colorectal, urothelial bladder and pancreatic cancer [16,17,21].

The monoclonal antibody has been previously described in detail [23]. In brief, mice were immunized with the undifferentiated human embryonic (hES) stem cell line SA167 (Cellartis, Göteborg, Sweden, www.cellartis.com). All animal experiments were performed in accordance with the Animal Welfare Ordinance and the Animal Welfare Act of Sweden and approved by the Animal Experiments Ethics Committee of Gothenburg (approval no. 310–2005).

By conventional hybridoma technology, hybridoma cell lines were established producing mAbs against hES cells. Mimotope analysis, immunoprecipitation, and mass-spectrometry identified the target antigen as PODXL. The epitopes of both the monoclonal and the polyclonal antibody are in the extracellular portion of PODXL. Of four protein-coding PODXL splice variants, the epitope sequence of the pAb matches three at 100% (PODXL 001, 005, and 201, The Human Protein Atlas). The fourth splice variant matches at 87% (PODXL 202). The epitope sequence of the mAb HES9 matches all splice variants 100%. [24,25]

### Immunohistochemistry

Tumor-tissue microarray blocks were freshly cut into 4-μm sections. After deparaffinization in xylene and rehydration through a gradually decreasing concentration of ethanol to distilled water, slides were treated in a PreTreatment module (Lab Vision Corp., Fremont, CA, USA) in Tris-Hcl (pH 8.5) buffer for 20 min at 98°C for antigen retrieval. Staining of sections was performed in an Autostainer 480 (Lab Vision Corp., Fremont, CA, USA) by the Dako REAL EnVision Detection system, Peroxidase/DAB+, Rabbit/Mouse (Dako, Glostrup, Denmark). Tissues were incubated with the mAb (dilution 1:800 = 8 μg/ml) and with pAb (dilution 1:250 = 2.5 μg/ml) for one hour at room temperature. A sample of colon tissue served as a positive control in each staining series.

### Evaluation of PODXL staining

PODXL expression by the mAb was evenly distributed in the cytoplasm and was often granular. By the pAb, PODXL expression was also cytoplasmic with no expression in the nuclei. In many cases, a distinct membranous positivity emerged, even in cells with weak cytoplasmic positivity. Cytoplasmic staining was scored as negative (0), weakly positive (1), moderately positive (2) or strongly positive (3) according to staining intensity. For cases with distinct membranous staining by the pAb, the score was recorded as 3, regardless of cytoplasmic staining intensity. The highest score of the triplicates of each sample was considered representative for analysis. The stainings were scored by two independent investigators (K.S. and J.H.) without knowledge of clinical data and outcome. Differing scores required determination of a consensus score.

### Statistical analyses

For statistical purposes, categories of PODXL expression were dichotomized for the mAb into low (0–2) and high (3) and for the pAb into non-membranous (0–2) and membranous (3). A categorization with three classes was created to study the two antibodies together: low (mAb, low and pAb, non-membranous), moderate (either mAb, high or pAb, membranous), and high (mAb, high and pAb, membranous). The Fischer exact-test or linear-by-linear association test served in evaluating associations between PODXL expression and relevant clinocopathological parameters. Survival analysis was done by the Kaplan-Meier method and compared by the log-rank test. The Bonferroni correction was used for multiple comparisons by dividing the probability level by the number of comparisons. The Cox regression proportional hazard model served for uni- and multivariate survival analysis adjusted for age, gender, stage, lymph-node ratio, and perivascular invasion. Interaction terms were considered, but with no significant interactions found. The Cox model assumption of constant hazard ratios over time was tested by including a time-dependent covariate separately for each testable variable at a time. The assumption was met with all variables. Variables with p<0.05 in univariate analysis (age, gender, T, N, stage, lymph node ratio (≥/<20%), grade, perineural invasion, perivascular invasion, mAb, pAb and combination of mAb and pAb) were selected into the multivariate model, retaining age and sex in all models. To simplify the model, stage and lymph node ratio (LNR) were combined into a single variable. All tests were two-sided. A p-value of 0.05 or less was considered significant. All statistical analyses were with SPSS version 20.0 (IBM SPSS Statistics, version 20.0 for Windows; SPSS, Inc., Chicago, IL, USA, an IBM Company).

## Results

### Immunohistochemical staining

PODXL expression by the pAb was cytoplasmic in tumor cells, but in some cases, distinct membranous expression was visible, not correlating with intensity of cytoplasmic expression. Such a staining pattern was not apparent for the mAb: it was cytoplasmic and evenly distributed.

**Fig 1:**
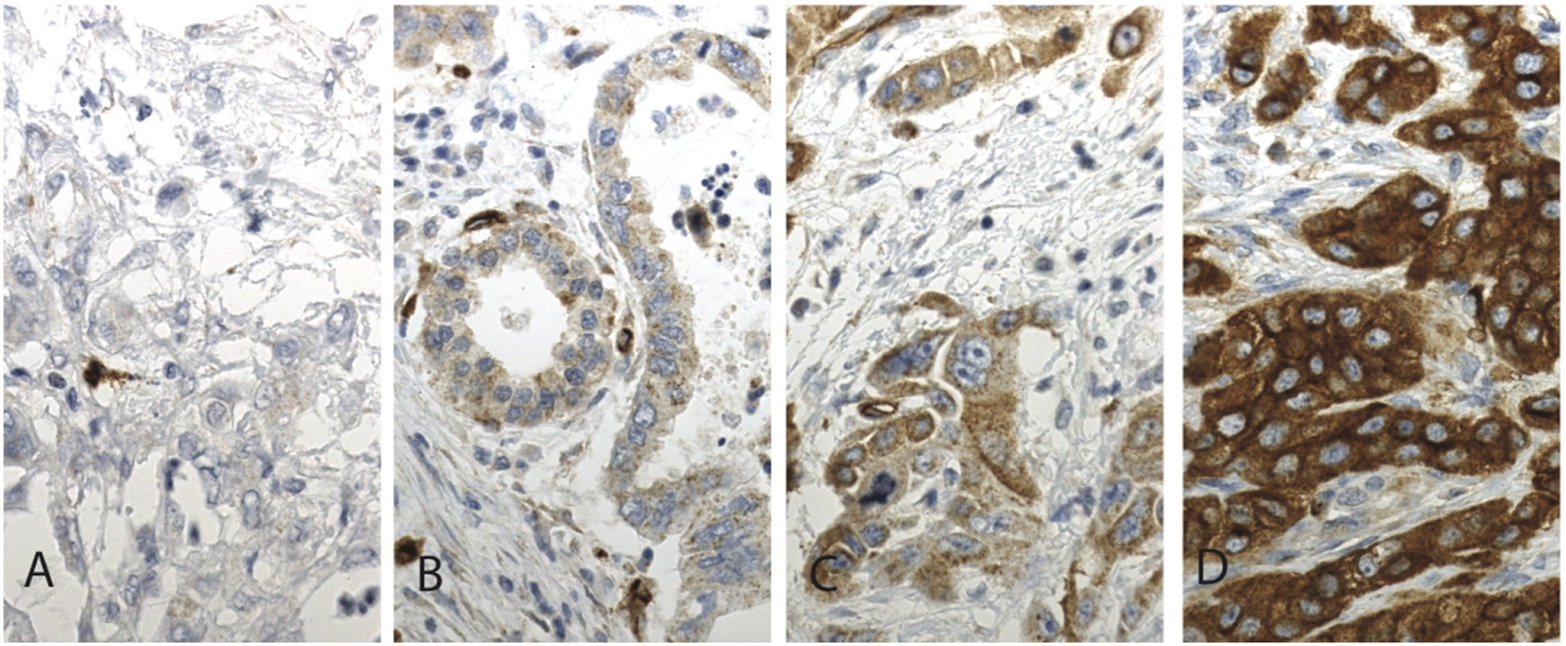
Immunohistochemical staining pattern of PODXL by polyclonal antibody HPA 2110 in pancreatic ductal adenocarcinoma. Representative images of PODXL expression in pancreatic ductal adenocarcinoma. (A) PODXL-negative, (B) weak cytoplasmic positivity, (C) moderate cytoplasmic positivity, and (D) strong and positive membranous positivity. Original magnification was x 400.

**Fig 2:**
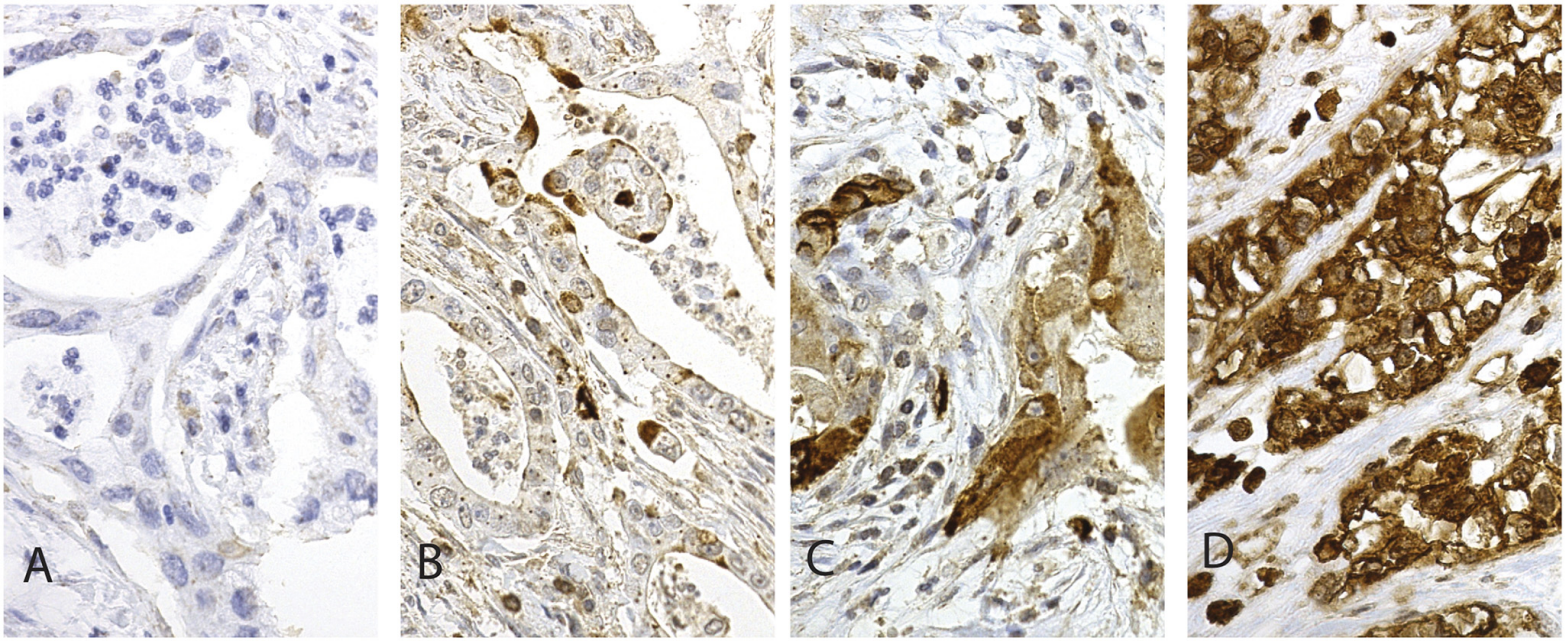
Immunohistochemical staining pattern of PODXL by monoclonal antibody HES9 in pancreatic ductal adenocarcinoma. Representative images of PODXL expression in pancreatic ductal adenocarcinoma. (A) PODXL-negative, (B) weak cytoplasmic positivity, (C) moderate cytoplasmic positivity, and (D) strong cytoplasmic positivity. Original magnification was x 400.

PODXL staining by the pAb could be evaluated in 166 (98.8%) specimens: 13 (7.8%) showing negative, 71 (42.8%) weak, 55 (33.1%) moderate, and 27 (16.3%) strong staining (Fig 1). PODXL staining by the mAb could be evaluated in 165 (98.2%) specimens: 21 (12.7%) showing negative, 69 (41.8%) weak, 39 (23.6%) moderate, and 36 (21.8%) strong staining (Fig 2).

### Association of PODXL expression with clinicopathological parameters separately and combined

An association emerged between membranous PODXL expression by the pAb and T (p = 0.045) and perineural invasion (p = 0.005). High PODXL expression by the mAb was associated with poor differentiation (p = 0.033). PODXL expression correlated neither with age, gender, stage, lymph-node ratio, tumor location (not shown), nor perivascular invasion (Tables 1 and 2).

**Table 1:** Association of clinicopathological parameters and PODXL expression by polyclonal antibody HPA 2110.

	Non-membranous	Membranous	
n(%)	93 (56.0)	73 (44.0)	p-value
**Age, years**			
<65	46 (49.5)	36 (49.3)	1.000
≥65	47 (50.5)	37 (50.7)	
**Gender**			
Male	52 (55.9)	40 (54.8)	1.000
Female	41 (44.1)	33 (45.2)	
**T**			
1	10 (11.0)	2 (2.7)	0.045
2	25 (27.5)	18 (24.7)	
3	54 (59.3)	50 (68.5)	
4	2 (2.2)	3 (4.1)	
Missing	2		
**N**			
0	26 (28.6)	23 (31.5)	0.733
1	65 (71.4)	50 (68.5)	
Missing	2		
**M**			
0	88 (95.7)	69 (95.8)	1.000
1	4 (4.3)	3 (4.2)	
Missing	1	1	
**Stage (WHO)**			
IA	7 (7.9)	2 (2.8)	0.606
IB	9 (10.1)	9 (12.3)	
IIA	10 (11.2)	10 (13.7)	
IIB	58 (65.2)	46 (63.0)	
III	0 (0.0)	3 (4.1)	
IV	5 (5.6)	3 (4.1)	
Missing	4		
**Lymph node ratio**			
<20%	68 (75.6)	56 (77.8)	0.852
≥20%	22 (24.4)	16 (22.2)	
Missing	3	1	
**Grade**			
1	17 (21.2)	7 (11.1)	0.103
2	53 (66.3)	44 (69.8)	
3	10 (12.5)	12 (19.1)	
Missing	13	10	
**Perineural invasion**			
Yes	51 (67.1)	57 (87.7)	0.005
No	25 (22.9)	8 (12.3)	
Missing	17	8	
**Perivascular invasion**			
Yes	23 (30.7)	26 (43.3)	0.151
No	52 (69.3)	34 (56.7)	
Missing	18	13	

Fischer exact-test was used for 2x2 tables and linear-by-linear association test for tables with more than two rows. Missing data excluded from the analyses.

**Table 2:** Association of clinicopathological parameters and PODXL expression by monoclonal antibody HES9.

	Low	High	
n(%)	129 (78.2)	36 (21.8)	p-value
**Age, years**			
<65	63 (48.8)	18 (50.0)	1.000
≥65	66 (51.2)	18 (50.0)	
**Gender**			
Male	73 (56.6)	18 (50.0)	0.570
Female	56 (43.4)	18 (50.0)	
**T**			
1	10 (7.9)	2 (5.7)	0.776
2	31 (24.4)	12 (34.3)	
3	83 (65.4)	21 (60.0)	
4	3 (2.3)	1 (2.9)	
Missing	2	1	
**N**			
0	38 (29.9)	11 (30.6)	1.000
1	89 (70.1)	25 (69.4)	
Missing	2		
**M**			
0	124 (96.9)	33 (94.3)	0.610
1	4 (3.1)	2 (5.7)	
Missing	1	1	
**Stage (WHO)**			
IA	7 (5.6)	2 (5.6)	0.531
IB	15 (12.0)	3 (8.3)	
IIA	16 (12.8)	4 (11.1)	
IIB	80 (64.0)	24 (66.6)	
III	2 (1.6)	1 (2.8)	
IV	5 (4.0)	2 (5.6)	
Missing	4		
**Lymph node ratio**			
<20%	96 (76.8)	27 (75.0)	0.826
≥20%	29 (23.2)	9 (25.0)	
Missing	4		
**Grade**			
1	20 (18.2)	4 (12.5)	0.033
2	78 (70.9)	18 (56.3)	
3	12 (10.9)	10 (31.2)	
Missing	19	4	
**Perineural invasion**			
Yes	80 (73.4)	27 (87.1)	0.151
No	29 (26.6)	4 (12.9)	
Missing	20	5	
**Perivascular invasion**			
Yes	32 (30.8)	15 (50.0)	0.081
No	72 (69.2)	15 (50.0)	
Missing	25	6	

Fischer exact-test was used for 2x2 tables and linear-by-linear association test for tables with more than two rows. Missing data excluded from the analyses.

Analysis of combined PODXL expression with clinicopathological parameters showed significant associations between high PODXL expression and poor differentiation (p = 0.014), perineural invasion (p = 0.007) and perivascular invasion (p = 0.039). High expression failed to correlate with age, gender, stage, or lymph node ratio (Table 3).

**Table 3:** Association of clinicopathological parameters and PODXL expression by polyclonal antibody HPA 2110 and monoclonal antibody HES9 combined.

	Low	Moderate	High	
n (%)	87 (53.0)	45 (27.4)	32 (19.6)	p-value
**Age, years**				
<65	43 (49.4)	22 (48.9)	16 (50.0)	1.000
≥65	44 (50.6)	23 (51.1)	16 (50.0)	
**Gender**				
Male	50 (57.5)	24 (53.3)	17 (53.1)	0.689
Female	37 (42.5)	21 (46.7)	15 (46.9)	
**T**				
1	10 (11.7)	0 (0.0)	2 (6.3)	0.227
2	22 (25.9)	12 (26.7)	9 (28.1)	
3	52 (61.2)	31 (68.9)	20 (62.5)	
4	1 (1.2)	2 (4.4)	1 (3.1)	
Missing	2			
**N**				
0	25 (29.4)	14 (31.1)	10 (31.3)	0.829
1	60 (70.6)	31 (68.9)	22 (68.7)	
Missing	2			
**M**				
0	83 (96.5)	44 (97.8)	29 (93.5)	0.602
1	3 (3.5)	1 (2.2)	2 (6.5)	
Missing	1		1	
**Stage (WHO)**				
IA	7 (8.4)	0 (0.0)	2 (6.3)	0.419
IB	8 (9.7)	8 (17.8)	2 (6.3)	
IIA	10 (12.0)	6 (13.3)	4 (12.5)	
IIB	54 (65.1)	28 (62.2)	21 (65.6)	
III	0 (0.0)	2 (4.5)	1 (3.1)	
IV	4 (4.8)	1 (2.2)	2 (6.3)	
Missing	4			
**Lymph node ratio**				
<20%	63 (75.0)	35 (79.5)	24 (75.0)	0.907
≥20%	21 (25.0)	9 (20.5)	8 (25.0)	
Missing	3	1		
**Gradus**				
1	16 (21.6)	5 (12.8)	3 (10.7)	0.014
2	51 (68.9)	28 (71.8)	17 (60.7)	
3	7 (9.5)	6 (15.4)	8 (28.6)	
Missing	13	6	4	
**Perineural invasion**				
Yes	48 (67.6)	32 (75.0)	26 (92.9)	0.007
No	23 (32.4)	8 (25.0)	2 (7.1)	
Missing	16	5	4	
**Perivascular invasion**				
Yes	19 (27.1)	15 (41.7)	13 (48.1)	0.039
No	51 (72.9)	21 (58.3)	14 (51.9)	
Missing	17	9	5	

Linear-by-linear association test was used for this table. Missing data excluded from the analyses.

### Survival analysis

**Fig 3:**
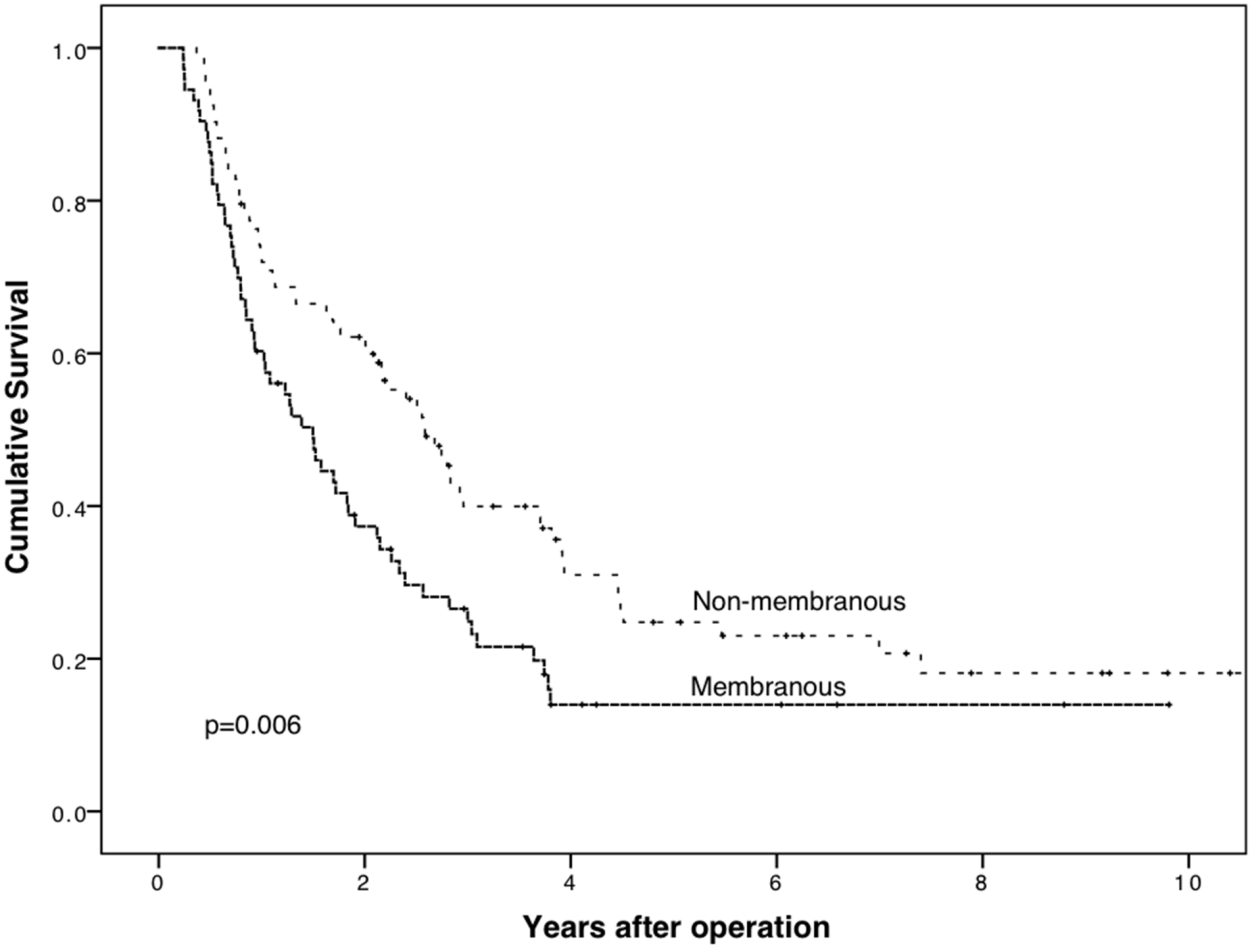
Membranous PODXL expression by polyclonal antibody HPA 2110 is a marker of poor prognosis in pancreatic ductal adenocarcinoma. Cancer-specific survival analysis according to the Kaplan-Meier method for membranous PODXL expression by the polyclonal antibody HPA 2110 in pancreatic ductal adenocarcinoma. Log-rank test was used here.

For PDAC patients with membranous PODXL expression by the polyclonal antibody, cancer-specific survival (CSS) was significantly worse (p = 0.006, Log-rank test); five-year CSS was 14.0% (95% CI 5.2–22.8%) for patients with membranous PODXL expression compared to 24.8% (95% CI 15.0–34.6%) for those with non-membranous expression (Fig 3).

**Fig 4:**
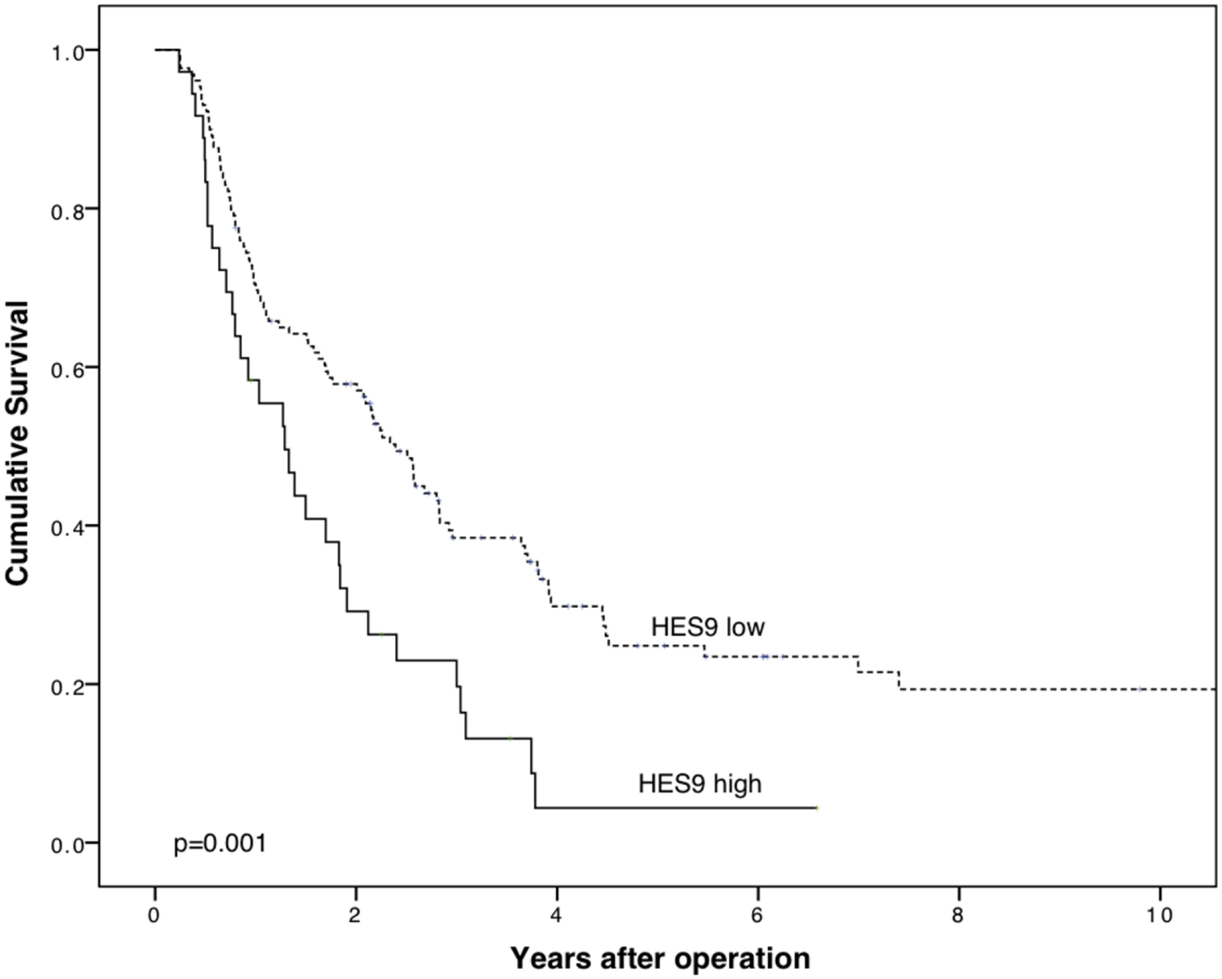
High PODXL expression by monoclonal antibody HES9 is a marker of poor prognosis in pancreatic ductal adenocarcinoma. Cancer-specific survival analysis according to the Kaplan-Meier method for high PODXL expression by the monoclonal antibody HES9 in pancreatic ductal adenocarcinoma. Log-rank test was used here. MAb HES9 recognizes PODXL protein.

PDAC patients with high PODXL expression by the monoclonal antibody showed a significantly poorer CSS than did those with low expression (p = 0.001); five-year CSS for PDAC patients with low expression was 24.8% (95% CI 16.4–33.2%) and for those with high expression, 4.4% (95% CI -3.6–12.4%) (Fig 4).

**Fig 5:**
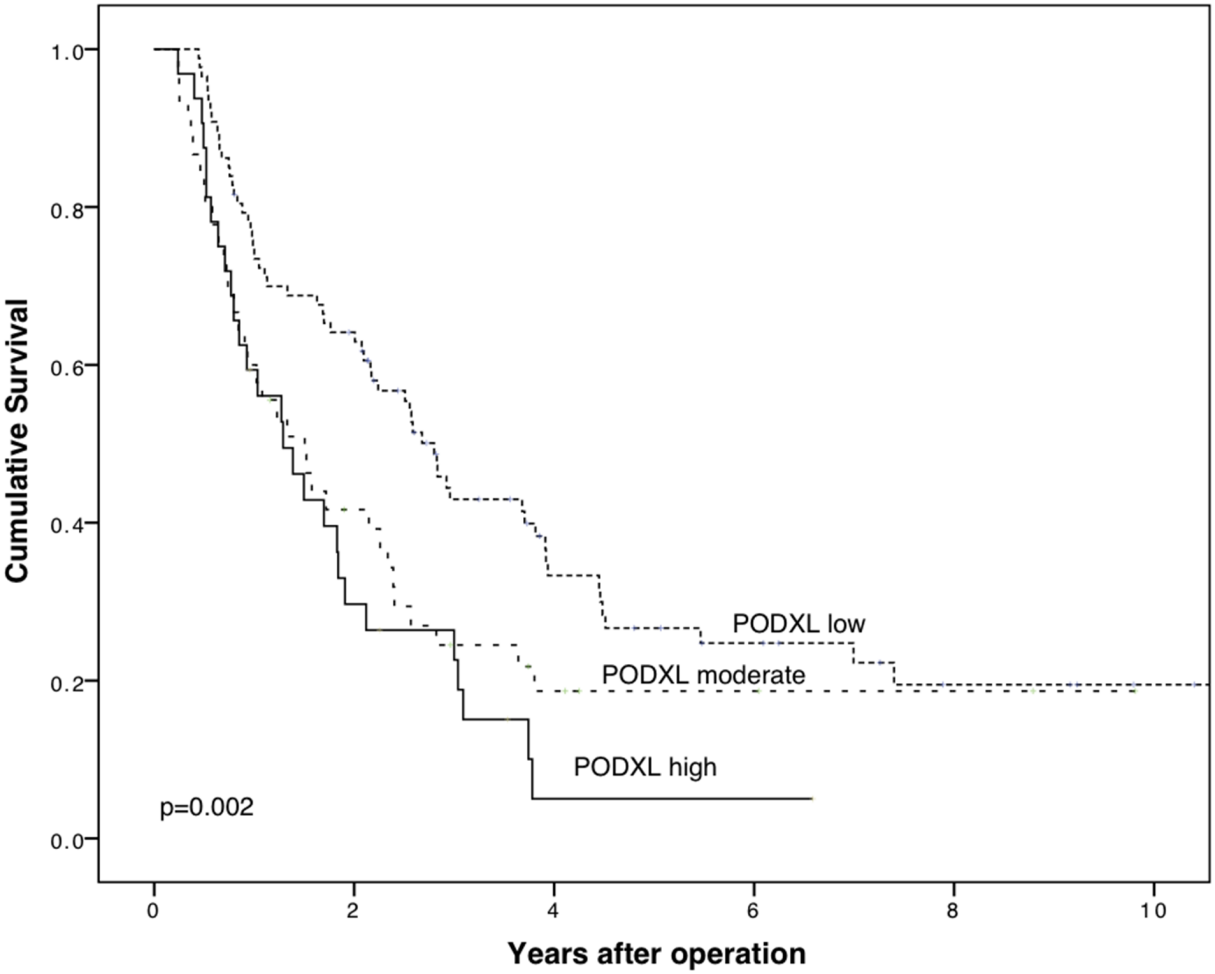
Concomitant positivity by two PODXL antibodies. Cancer-specific survival analysis according to the Kaplan-Meier method for combined expression of PODXL by the polyclonal antibody HPA 2110 and the monoclonal antibody HES9. A categorization with three classes was created to study the two antibodies together: low (mAb, low and pAb, non-membranous), moderate (either mAb, high or pAb, membranous), and high (mAb, high and pAb, membranous).

A combination of mAb and pAb showed a significantly poorer CSS for PDAC patients with high expression than for those with low expression (p = 0.001). No significant difference in CSS appeared, however, between patients with moderate and high expression (p = 0.37), or between moderate and low expression (p = 0.020, Bonferroni correction used). Five-year CSS for patients with low expression was 26.6% (95% CI 16.2–37.0%), with moderate expression 18.7% (95% CI 6.5–30.9%) and with high expression 5.0% (95% CI -4.2–14.2%) (Fig 5).

Cox regression univariate analysis confirmed these results. High podocalyxin expression associated significantly with higher risk of death from PDAC by both the pAb (HR = 1.62; 95% CI 1.12–2.33; p = 0.01) and the mAb (HR = 2.10, 95% CI 1.38–3.20; p<0.001). The combined moderate expression of PODXL by both the mAb and pAb showed higher risk of death from PDAC (HR = 1.56, 95% CI 1.02–2.40; p = 0.042), and the risk was even higher with combined high expression (HR = 2.21, 95% CI 1.39–3.54; p<0.001). In multivariate analyses, adjusted for age, gender, stage, lymph-node ratio, and perivascular invasion, membranous PODXL expression by the pAb and high PODXL expression by the mAb remained statistically significant. The combined moderate and high expression of PODXL by both mAb and pAb also remained, by multivariate analysis, statistically significant (Table 4).

**Table 4:** Cox uni- and multivariate analysis of relative risk of death from pancreatic ductal adenocarcinoma by PODXL expression.

Polyclonal antibody			Monoclonal antibody			Combined		
PODXL expression	HR (95% CI)	P-value	PODXL expression	HR (95% CI)	P-value	PODXL expression	(HR 95% CI)	P-value
	**Univariate**			**Univariate**			**Univariate**	
Non-membranous	1.00		Low	1.00		Low	1.00	
Membranous	1.62 (1.12–2.33)	0.01	High	2.10 (1.38–3.20)	<0.001	Moderate	1.56 (1.02–2.40)	0.042
						High	2.21 (1.39–3.54)	<0.001
	**Multivariate**			**Multivariate**			**Multivariate**	
Non-membranous	1.00		Low	1.00		Low	1.00	
Membranous	2.03 (1.32–3.13)	0.001	High	2.36 (1.47–3.80)	<0.001	Moderate	2.07 (1.25–3.44)	0.005
						High	2.67 (1.55–4.59)	<0.001

Abbrevations CI = Confidence interval, HR = Hazard ratio. Multivariate analysis included adjustment for age, gender, stage (IA-IIA, IIB and III), lymph node ratio (≥/< 20%), and perivascular invasion.

## Discussion

We demonstrate that in PDAC tissue overexpression of PODXL is an independent predictor of poor prognosis, and that it associates with unfavorable clinicopathological factors. To our knowledge, this is the first report of the prognostic value of PODXL expression in PDAC.

PODXL expression has been demonstrated in PDAC earlier [21,22]. Ney et al reported that PODXL expression differentiates PDAC from adenocarcinomas occurring in the biliary tract [21]. In their study, 44% of the PDAC specimens were positive for PODXL. Dallas et al [22] showed immunopositivity for PODXL in 69% of the PDAC specimens. Staining intensity was reported in neither study. In our series, immunopositivity for PODXL was more frequent: 92.2% of the specimens by the pAb and 87.3% by the mAb. One explanation could be differences in staining technique, in evaluation of the specimens, or in choice of cut-off points. In our study both the polyclonal and the monoclonal antibody used show a similar proportion of positivity. The antibody used in the study of Ney’s group differed from ours, whereas Dallas’ group used the same polyclonal antibody as we did.

We also show that immunostaining of PODXL by our new in-house mAb gives prognostic results similar to those achieved by the commercial polyclonal antibody (HPA 2110, Atlas Antibodies, Stockholm, Sweden) in colorectal cancer [15,16]. Our results also are in line with the studies of colorectal cancer by the same mAb [23,26].

It has been shown that, in cancer, poor prognosis associates with distinct membranous PODXL expression, rather than cytoplasmic expression [15,27]. These findings are substantiated by earlier studies demonstrating that aberrant PODXL expression supports the disruption of cell-to-cell and cell-to-extracellular matrix adhesions, and in this way promotes tumor dissemination [28]. In cancer cells the staining pattern of the monoclonal antibody was different, with mainly cytoplasmic expression in cancer cells with no distinct membranous immunopositivity. The reason for this is unknown. In PDAC, the proportion of tumors with high cytoplasmic or membranous PODXL expression was relatively large (21.8% and 44.0%, respectively) compared to corresponding percentages in studies of colorectal [15], breast [14], or urothelial bladder cancer [17].

The two antibodies used in this study recognize different epitopes in the extracellular region of the PODXL molecule. This may explain why their expression patterns varied, and their case-by-case expressions differed. Kaprio et al. hypothesized in the study of CRC that the pAb recognizes an active form of PODXL at the cell membrane, whereas the mAb recognizes overexpression of cytoplasmic PODXL [26]. In CRC, a subgroup with even worse prognosis was identified when the mAb expression and the pAb expression of PODXL both were high and membranous [26]. Here, similar evidence for PDAC was found.

Those patients, who had received neoadjuvant chemotherapy, were excluded from the study, because it is not known how the chemotherapy would affect the tumor specimens. Only a small difference existed between the two antibodies as prognostic markers: the hazard ratio for risk of death was greater for patients with high PODXL expression by the mAb than for those with membranous PODXL expression by the pAb. However, PODXL remained an independent prognostic factor in multivariate analysis independent of the antibody. Scoring was easier and staining differences were clearer by the mAb than by the pAb. Five-year CSS was lower for patients with high PODXL expression by the mAb than for patients with membranous PODXL expression by the pAb (4.4% vs. 14.0%). This supports the role of cytoplasmic overexpression of PODXL as a marker of poor prognosis.

The strength of this study is a well-characterized, consecutive, and quite large patient cohort with PDAC. Clinical follow-up and survival data were reliable and up-to-date. All histological samples were re-evaluated by experienced pathologists; other types of pancreatic cancer other than ductal adenocarcinoma were excluded. By the TMA technique, only a small area of each tumor is evaluated and thus the staining result may not be representative. We tried to diminish this possible error by taking cores from different parts of the tumor and altogether scored six different spots for each tumor. Only a small proportion (1.2–1.8%) of our TMA spots were lost due to technical reasons.

## Conclusion

To our knowledge, this is the first report to show that PODXL is an independent marker of poor prognosis in PDAC. Both membranous expression of PODXL by a polyclonal antibody (HPA 2110) and high cytoplasmic expression of PODXL by a monoclonal antibody (HES9) defined groups with poor prognosis. To clarify the different expression patterns of the two antibodies, we can speculate that they can either recognize different variants of PODXL in PDAC or identify PODXL at different form in the cytoplasm or cellular membrane.
